# Rheumatoid arthritis-associated lung disease in black Africans: Descriptive study of 28 cases in Lomé

**DOI:** 10.7196/AJTCCM.2020.v26i4.074

**Published:** 2020-12-01

**Authors:** A G Gbadamassi, K S Adjoh, A N E Fianyo, T A S Adambounou, A K Aziagbe, P Efalou

**Affiliations:** 1 Pulmonology Unit, Department of Medicine, Sylvanus Olympio Hospital, University of Lomé, Togo; 2 Rheumatology Unit, Department of Medicine, Sylvanus Olympio Hospital/Regional Hospital/District III Hospital,University of Lomé, Togo

**Keywords:** Rheumatoid arthritis, Lung disease, Black Africans, Lomé

## Abstract

**Background:**

Several studies have shown that lung disease is a common extra-articular manifestation of rheumatoid arthritis (RA).

**Objectives:**

To describe the lung manifestations in the RA population in Lomé, Togo.

**Methods:**

The study was conducted from October 2018 to July 2019 at the pulmonology unit of the Sylvanus Olympio University teaching hospital, in collaboration with rheumatology centres in Lomé, Togo. Patients meeting the American College of Rheumatology criteria for RA were prospectively enrolled. They underwent clinical examination, spirometry, a 6-minute walk test (6MWT) and a chest X-ray (CXR). All information collected and surveys gathered were subjected to statistical analysis.

**Results:**

Twenty-four out of 28 patients were women (85.7%). The mean (standard deviation (SD)) duration of illness was 4.1 (2.8) years. Thirteen patients out of 28 (46.4%) had respiratory symptoms. On CXR, interstitial lung disease was the only pleuropulmonary lesion (17.8%). Spirometry was abnormal in 25% of cases, with a predominance of restrictive ventilatory disorder (21.4%). The 6MWT was abnormal in 25% of patients. A total of 20 patients (71.4%) had at least one lung manifestation. We noted that there were significantly more patients with respiratory symptoms and no radiographical abnormalities than those with both respiratory symptoms and radiographical abnormalities (p=0.013).

**Conclusion:**

Lung changes affect a significant proportion of RA patients in Lomé. Studies conducted with appropriate respiratory investigations and combining cardiovascular explorations will bring us closer to an understanding of the effects of RA-associated lung disease.

## Background

Rheumatoid arthritis (RA) is a systemic inflammatory disorder of unknown pathogenesis that most often affects the joints, resulting in progressive, symmetrical and erosive destruction of cartilage and bone. It is the most common connective tissue disease,^[1]^ affecting ~1% of the white populations in North America and Europe.^[2]^ The prevalence of RA in black Africans is considered low, but is difficult to quantify.^[3,4]^

Although it appears primarily as a joint disease, RA is a systemic condition that can affect several organs, including the heart, lungs, kidneys, eyes, nerves and skin. One study revealed extra-articular manifestations of RA in a quarter of patients,^[2]^ while another showed a cumulative incidence of 15 years in 53% of them.^[2]^ Of these, lung disease is the common extra-articular manifestation of RA detected in 40 - 70 % of cases^[2,5]^ and a major cause of morbidity and mortality in RA patients.^[6]^ In some cases, respiratory symptoms may precede the development of joint symptoms or the diagnosis of RA.^[7]^ This may involve all parts of the lung, including pleura, airways, parenchyma and vasculature.^[8]^ These changes may reflect chronic immune activation, increased susceptibility to infection (often related to immunomodulatory medications) or direct toxicity due to disease-modifying or biological therapy.^[1,9]^

Few studies on RA-associated lung diseases have been conducted in black Africans,^[10,11]^ while many have been conducted in North American and European white populations.^[6,8,12]^ The objective of this study was to describe the lung manifestations in the RA population of Lomé, Togo.

## Methods

### Patients

Patients meeting the American College of Rheumatology (ACR) criteria for RA^[13]^ were included in this study, regardless of disease duration and the presence of pulmonary symptoms. They were selected from the outpatient pulmonology clinic of the Sylvanus Olympio University hospital of Lomé, and from rheumatology clinics in three central hospitals in Lomé (Sylvanus Olympio University hospital, regional hospital and district III hospital). They were collected over a period from October 2018 to July 2019, and informed oral consent was obtained from each participating patient. All patients underwent clinical assessment, spirometry test, a 6-minute walk test (6MWT) and a chest X-ray (CXR).

### Clinical Assessment

A full clinical evaluation of the chest was performed to look for any symptoms or signs suggesting lung disease. Other extra-articular symptoms or signs were also noted.

### Pulmonary function tests (PFT's)

Spirometry was obtained with Spirolab MIR. It was performed in accordance with the guidelines of the American Thoracic Society and European Respiratory Society. ^[14]^ Vital capacity, forced vital capacity (FVC), forced expiratory volume in 1 second (FEV1), FEV1/FVC ratio with a threshold 70%, total lung capacity and maximum expiratory flow at 25% of vital capacity, 50% of vital capacity and 75% of vital capacity were found in patients tested in a seated position. The measurements were obtained without the use of bronchodilators.

A 6MWT was obtained with Cosmed Spiropalm. The 6MWT was abnormal when there was desaturation of 4% and more compared with the initial saturation. Patients who did not undergo a 6MWT due to joint disease were not included in the evaluation of the 6MWT results. Those who did not undergo PFTs within 2 months of the standard CXR were also excluded from the PFTs outcome evaluation.

The plethysmography and carbon monoxide diffusing capacity (DLCO) tests were not performed due to their absence in our setting.

### Chest X-ray assessment

Chest pain standard radiography was obtained with the YSX500D 500 mA digital X-ray machine. A radiologist and a pulmonologist agreed after discussing each patient’s final report. The lung lesion site on the CXR was distributed based on the international classification pneumoconiosis X-rays of the International Labor Office (ILO):^[15]^ upper-right area (URA), middle-right area (MRA), lower-right area (LRA), upper-left area (ULA), middle-left area (MLA), lower left area (LLA).

High-resolution chest computed tomography (HRCT) was not performed owing to its unavailability in our setting.

### Statistical Analysis

Data were entered using EpiData 3.1 and analysed using STATA version 14 (StataCorp., USA). The χ2 test (or Fisher’s exact test if the numbers were <5) was used to compare the categorical variables. The significance limit was set at 0.05.

## Results

The study considered 28 RA cases, including 4 men (14.3%) and 24 women (85.7%). The male:female ratio was 1:6. The mean age of male patients was 35.2 (6.2) years, that of female patients was 46.8 (17.0) years and that of all patients was 45.2 (16.4) years, with extremes of 10 and 75 years. With respect to age, the majority of patients (57.1%) were younger than 49 years. Half of them (50%) had a duration of illness between 1 and 3 years. The mean duration of illness was 4.1 (2.8) years, with extremes of 1 and 12 years. High blood pressure was the most common medical history (14.3%). As an immunological assessment, only antinuclear antibodies were found in 5 patients (17.8%). They were high in 1 patient (20%) and normal in 4 (80%). Rheumatoid factor and anticitrullinated protein antibodies were not found in any patient. All patients were on anti-RA therapy at diagnosis. Anti-RA drugs were administered with no respiratory examination. The majority of patients (71.4%) were taking oral prednisone associated with methotrexate (MTX), 4 (14.3%) only received non-steroidal anti-inflammatory drugs (NSAIDs), 2 (7.1%) took only MTX, 1 (3.6%) took only prednisone and 1 (3.6%) was given only salazopyrin.

Thirteen of the 28 patients had respiratory symptoms (46.4%), including shortness of breath (61.5%), dry cough (38.5%), chest pain (30.7%) and sputum production (7.7%). Under no circumstances did respiratory symptoms precede the diagnosis of RA. Only three patients (10.7%) had clinical evidence of lung involvement in the form of bronchitis and crackles. No other extra-articular symptoms were noted.

In our study, all patients underwent a CXR: 5 (17.8%) had interstitial lung disease (ILD) and none were diagnosed with pleural abnormality. ILD affected lung URA in 2 cases (40%), MRA in 3 cases (60%), LRA in 5 cases (100%), ULA in 3 cases (60%), MLA in 3 cases (60%) and LLA in 4 cases (80%). ILD was bilateral in 4 cases (80%) and localised in the lower lobe in all cases. Apart from lung damage, 8 patients suffered from cardiomegaly (28.5%). One case of cardiomegaly was associated with ILD.

Spirometry was abnormal in 7 of the 28 patients (25%): 1 case of obstructive ventilatory disorder and 6 cases of restrictive ventilatory disorder. Abnormalities in the 6MWT were detected in 7 (25%) of the 28 patients.

Of the 28 patients, 8 had no abnormality in any of the parameters related to lung involvement. Consequently, a total of 20 patients (71.4%) had at least a lung involvement.

We noticed that patients with respiratory symptoms and no radiographical abnormality were significantly more numerous than those with both respiratory symptoms and radiographical abnormalities (Table 1). No other factor was significantly associated with chest radiographical abnormalities (Table 2). Patients aged ≥50 years had significantly longer RA duration than those of lower age (Table 3).

## Discussion

As shown in our study, RA is a disease with at least a 3:1 predilection for women between 20 and 50 years of age.^[16]^ It is often treated with prednisone or methotrexate.^[17,18]^

In RA-associated lung diseases, respiratory symptoms, usually insidious, include dyspnoea on exertion, and a non-productive cough.^[19]^ These symptoms are commonly seen in ILD, which is common in RA.^[18]^ This explains why bibasilar crackles are found in most patients in various studies^[19]^. However, it is currently estimated that ~30% of patients with RA have subclinical ILD noted on chest high-resolution computer tomography (HRCT).^[8]^ Also, recognition of exertional dyspnoea may be delayed due to exercise limitations associated with joint disease. As in our study, chest pain, which is often a sign of pleural disease, is less present. Pleural disease is also common in RA patients, but only 3 - 5% of patients are symptomatic.^[8]^

Several studies revealed that HRCT is much more sensitive than CXR in the evaluation of ILD, and that its higher sensitivity should allow early diagnosis.^[20]^ HRCT was not used in our study, which constitutes a limitation. However, the CXR also allowed us to realise that interstitial bibasilar lesions were the most common. The prevalence of ILD in RA patients is about 20 - 50% depending on the study method.^[19,21]^ Concerning pleural disease, pleural thickening and/or effusion was found in between 16 and 24% in chest radiography of RA patients.^[19]^ Also, RA-associated cardiovascular^[22]^ disease could explain the relatively high prevalence of cardiomegaly (28.5%) in our study.

One-third of RA patients usually have abnormal pulmonary function tests (PFTs).^[5]^ Habib et al.^[5]^ found that 32.5% of patients in their study had abnormal PFTs. This rate was slightly different from ours (25%), most likely due to the systematic use of lung diffusing capacity for carbon monoxide (DLCO) in their study. The majority of patients with RA-ILD will have a restrictive pattern on PFTs, with or without decreased DLCO and hypoxaemia.^[23]^ Decrement of FVC and DLCO is associated with a poorer prognosis. However, a study by Assayag et al.^[6]^ revealed that only decreased DLCO was statistically significant. PFTs were generally poor predictors of CXR and HRCT findings.^[5]^

More than two-thirds (71.4%) of our patients had RA-associated lung disease, contrasting with the results of Adelowo et al.,^[3]^ where no respiratory manifestation was found in RA patients. This difference is certainly due to the lack of systematic searching for respiratory disorders in the study conducted by Adelowo et al.^[3]^ The prevalence of pulmonary abnormalities in RA patients varies according to the characteristics of the study population, the definition of lung disease used, and the sensitivity of the clinical investigations used.^[19]^ In unselected populations, up to one-third of subjects describe important respiratory symptoms, but two-thirds or more may have significant radiographic abnormalities on HRCT.^[24]^

The present study showed that patients with respiratory symptoms and no radiographical abnormalities were significantly more numerous than those with both respiratory symptoms and radiographical abnormalities. This could be explained by the fact that the respiratory symptoms (dyspnoea and cough) are not specific for pulmonary damage, but may also be of cardiovascular origin.^[22]^ Indeed, eight patients (28.5%) had cardiomegaly in our study. Such differences are less important in studies where chest HRCT was systematic^[5]^ because the chest HRCT is much more sensitive and specific than CXR in the evaluation of ILD,^[20]^ and ILD is the most common pulmonary manifestation of RA-associated lung disease.^[18]^

## Conclusion

The results of our study indicate that lung changes affect a significant proportion (71.4%) of RA patients in Lomé, with a predominance of ILD. These conditions mostly occur in the first 4 - 5 years of the disease, as revealed in a study by Marigliano et al.^[12]^ This prevalence is undermined by the fact that our study lacks more specific explorations. Studies with appropriate investigations such as chest HRCT, plethysmography, DLCO and cardiovascular explorations will bring us closer to the reality.

## Figures and Tables

**Table:** Table 1. Factors influencing respiratory symptoms

	Respiratory symptoms	
	No, *n* (%)	Yes, *n* (%)	*p*-value
Gender			1
Male	2 (13.4)	2 (15.4)	
Female	13 (86.6)	11 (84.6)	
Age (years)			0.872
≤49	8 (53.3)	8 (61.5)	
50 - 64	5 (33.3)	3 (23)	
>65	2 (13.4)	2 (15.5)	
CXR abnormalities			0.013
No	15 (100)	8 (61.5)	
Yes	0	5 (38.5)	
Obstructive ventilatory disorder			0.464
No	15 (100)	12 (92.3)	
Yes	0	1 (7.7)	
Restrictive ventilatory disorder			1
No	12 (80)	10 (77)	
Yes	3 (20)	3 (23)	
Six-minute walk test abnormalities			1
No	10 (66.6)	8 (61.5)	
Yes	5 (33.4)	5 (38.5)	

CXR = chest X-ray

**Table:** Table 2. Factors influencing CXR abnormalities

	CXR abnormalities	
	No, *n* (%)	Yes, *n* (%)	*p*-value
Gender			1
Male	4 (17.4)	0	
Female	19 (82.6)	5 (100)	
Age (years)			0.057
≤49	15 (65.2)	1 (20)	
50 - 64	6 (26)	2 (40)	
>65	2 (8.8)	2 (40)	
Obstructive ventilatory disorder			1
No	22 (95.6)	5 (100)	
Yes	1 (4.4)	0	
Restrictive ventilatory disorder			0.285
No	19 (82.6)	3 (60)	
Yes	4 (17.4)	2 (40)	
Six-minute walk test abnormalities			0.315
No	16 (69.5)	2 (40)	
Yes	7 (30.5)	3 (60)	

CXR = chest X-ray

**Table:** Table 3. Factors influencing the duration of RA progression

	Duration of RA progression	
	No, *n* (%)	Yes, *n* (%)	*p*-value
Gender			1
Male	0	4 (16)	
Female	3 (100)	21 (84)	
Age (years)			0.032
≤49	0	16 (64)	
50 - 64	3 (100)	5 (20)	
>65	0	4 (16)	
Respiratory symptoms			0.087
No	0	15 (60)	
Yes	3 (100)	10 (40)	
Obstructive ventilatory disorder			1
No	3 (100)	24 (96)	
Yes	0	1 (4)	
Restrictive ventilatory disorder			0.107
No	1 (33.3)	21 (84)	
Yes	2 (66.7)	4 (16)	
Six-minute walk test abnormalities			1
No	2 (66.7)	16 (64)	
Yes	1 (33.3)	9 (36)	
CXR abnormalities			0.073
No	1 (33.3)	22 (88)	
Yes	2 (66.7)	3 (12)	

RA = rheumatoid arthritis

CXR = chest X-ray
